# Delayed Caecal Perforation Following Endoscopic Retrograde Cholangiopancreatography (ERCP)-Induced Severe Acute Pancreatitis: A Rare Pancreatitis Complication

**DOI:** 10.7759/cureus.102767

**Published:** 2026-02-01

**Authors:** Rami Ayoub, Konstantinos Baronos, Sara Arshad, Alice Millard, Marios Alogakos, Omaymah Al-Shweiki, Ashley Dennison, Neil Bhardwaj

**Affiliations:** 1 Department of Hepatopancreatobiliary Surgery, University Hospitals of Leicester NHS Trust, Leicester, GBR; 2 Department of Hepatobiliary Surgery, Al-Balqa Applied University, Al-Balqa, JOR; 3 Department of General Surgery, University Hospitals of Leicester NHS Trust, Leicester, GBR; 4 Department of Gastrointestinal Surgery, University Hospitals of Leicester NHS Trust, Leicester, GBR; 5 Department of Surgery, Indiana University School of Medicine, Indiana, USA; 6 Department of Endocrinology, University Hospital Coventry and Warwickshire, Coventry, GBR

**Keywords:** caecal perforation, endoscopic retrograde cholangiopancreatogram (ercp), pancreatitis, post-endoscopic retrograde cholangiopancreatography pancreatitis, post-ercp pancreatitis (pep)

## Abstract

Severe acute pancreatitis represents a systemic inflammatory condition that may extend beyond the pancreas and result in significant extra-pancreatic morbidity. Although colonic involvement is a recognised complication of severe or necrotising pancreatitis, it is uncommon and most frequently affects bowel segments adjacent to the pancreatic bed, with distal colonic perforation remaining exceedingly rare. Endoscopic retrograde cholangiopancreatography (ERCP) is a recognised iatrogenic trigger of acute pancreatitis and, in rare cases, may precipitate severe disease. We report a unique case of delayed caecal perforation developing fifteen days after the onset of severe post-ERCP pancreatitis (PEP) in a 64-year-old woman. The initial presentation of PEP was complicated by haemodynamic instability and multiorgan failure requiring prolonged intensive care unit support. Despite an initial period of apparent stabilisation, the patient subsequently deteriorated with gastrointestinal bleeding and haemodynamic compromise. Cross-sectional imaging demonstrated pneumoperitoneum arising from the caecum, and emergency laparotomy confirmed caecal perforation, which was managed with extended right hemicolectomy and end ileostomy formation. Histopathological examination demonstrated acute transmural ischaemic necrosis with associated omental fat saponification, in the absence of mechanical or primary vascular pathology, supporting an inflammatory and ischaemic mechanism secondary to severe acute pancreatitis. After a prolonged hospital stay and clinical stabilisation, the patient was discharged. This case highlights a rare and delayed manifestation of pancreatitis-associated colonic injury at a remote anatomical site and underscores the importance of maintaining a high index of suspicion for secondary gastrointestinal complications in patients with severe pancreatitis and unexplained clinical deterioration.

## Introduction

Acute pancreatitis is a common inflammatory condition with a broad clinical spectrum, ranging from mild, self-limiting disease to severe necrotising pancreatitis associated with significant morbidity and mortality [1]. Endoscopic retrograde cholangiopancreatography (ERCP) represents a recognised iatrogenic trigger of acute pancreatitis and remains an important cause of post-procedural pancreatic inflammation. Post-ERCP pancreatitis (PEP) occurs in approximately 2-10% of cases, with up to 0.5% progressing to severe disease associated with substantial morbidity and/or mortality [1-4]. Although gastrointestinal perforation has been reported following ERCP, with an estimated incidence of 0.1-0.6%, such injuries are most commonly attributed to mechanical factors, particularly stent migration, rather than pancreatitis itself causing colonic perforation [5-7].

Colonic perforation arising in the context of acute pancreatitis most frequently involves bowel segments in close anatomical proximity to the pancreatic bed, particularly the transverse colon and splenic flexure [8]. These complications are thought to result from the intense inflammatory response triggered by pancreatic injury, leading to endothelial dysfunction, microvascular thrombosis, and regional hypoperfusion, with extension of injury to adjacent segments of the gastrointestinal tract. Colonic involvement is a recognised but uncommon complication of severe or necrotising pancreatitis, encompassing a spectrum of manifestations including inflammation, stricturing, fistulation, ischaemia, necrosis, and, far less commonly, perforation. More distal colonic involvement, including isolated caecal perforation, is exceedingly rare and remains sparsely described in the literature [5-8]. The proposed mechanism involves retroperitoneal third spacing of activated pancreatic enzymes and inflammatory cytokines, which may track along the right paracolic gutter, resulting in localised chemical inflammation of serosal membranes, ischaemia, and eventual transmural necrosis and perforation [9].

In this report, we outline a unique case of delayed caecal perforation following severe PEP in a female patient who underwent ERCP for biliary stent removal. Such reports remain exceedingly rare in the literature, particularly involving the cecum. Accordingly, this case is presented to highlight the diagnostic and therapeutic challenges associated with pancreatitis-induced bowel injury occurring at an unexpected and remote anatomical site. It underscores the importance of maintaining a high index of suspicion for secondary gastrointestinal complications in patients with severe PEP and evolving abdominal sepsis, as delayed recognition of such complications may result in significant morbidity and necessitate extensive surgical intervention.

## Case presentation

A 64-year-old Caucasian woman with a past medical history limited to hypertension and osteoarthritis presented within hours of undergoing ERCP with acute severe epigastric abdominal pain, persistent bilious vomiting, and inability to tolerate oral intake. On initial assessment, she was acutely unwell and haemodynamically unstable, with hypotension (79/68 mmHg) and tachycardia (105 beats per minute).

**Figure 1:**
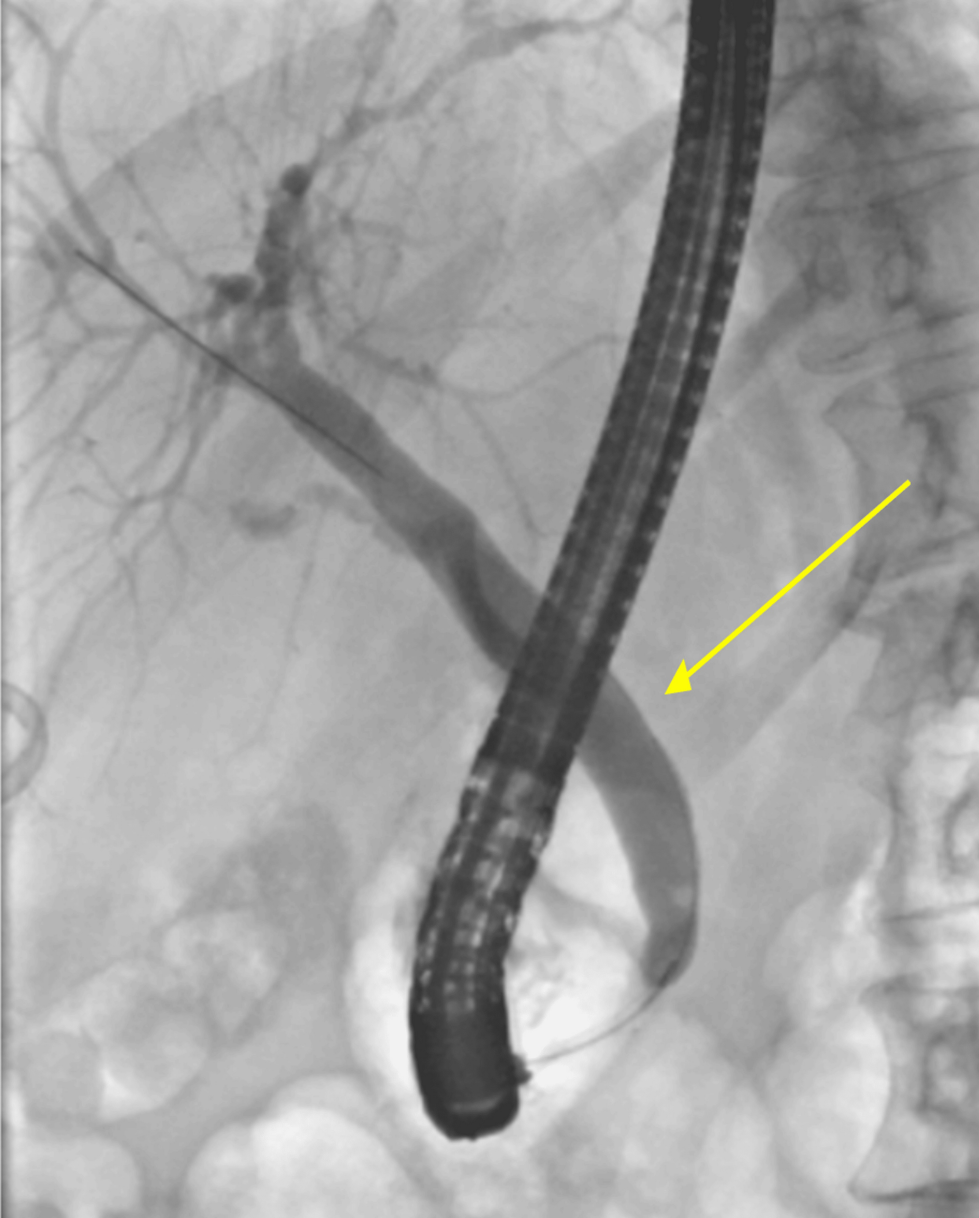
Cholangiogram after endoscopic retrograde cholangiopancreatography (ERCP) stent removal (yellow arrow).

Surgical history was significant for a laparoscopic cholecystectomy performed five months earlier, which was complicated by a cystic duct stump bile leak. This was managed with ERCP and insertion of a temporary biliary stent. Follow-up computed tomography (CT) scan demonstrated resolution of the bile leak. She subsequently underwent a planned ERCP for stent removal 14 weeks after the stent insertion and a cholangiogram (Figure 1), following which she became acutely unwell.

**Figure 2:**
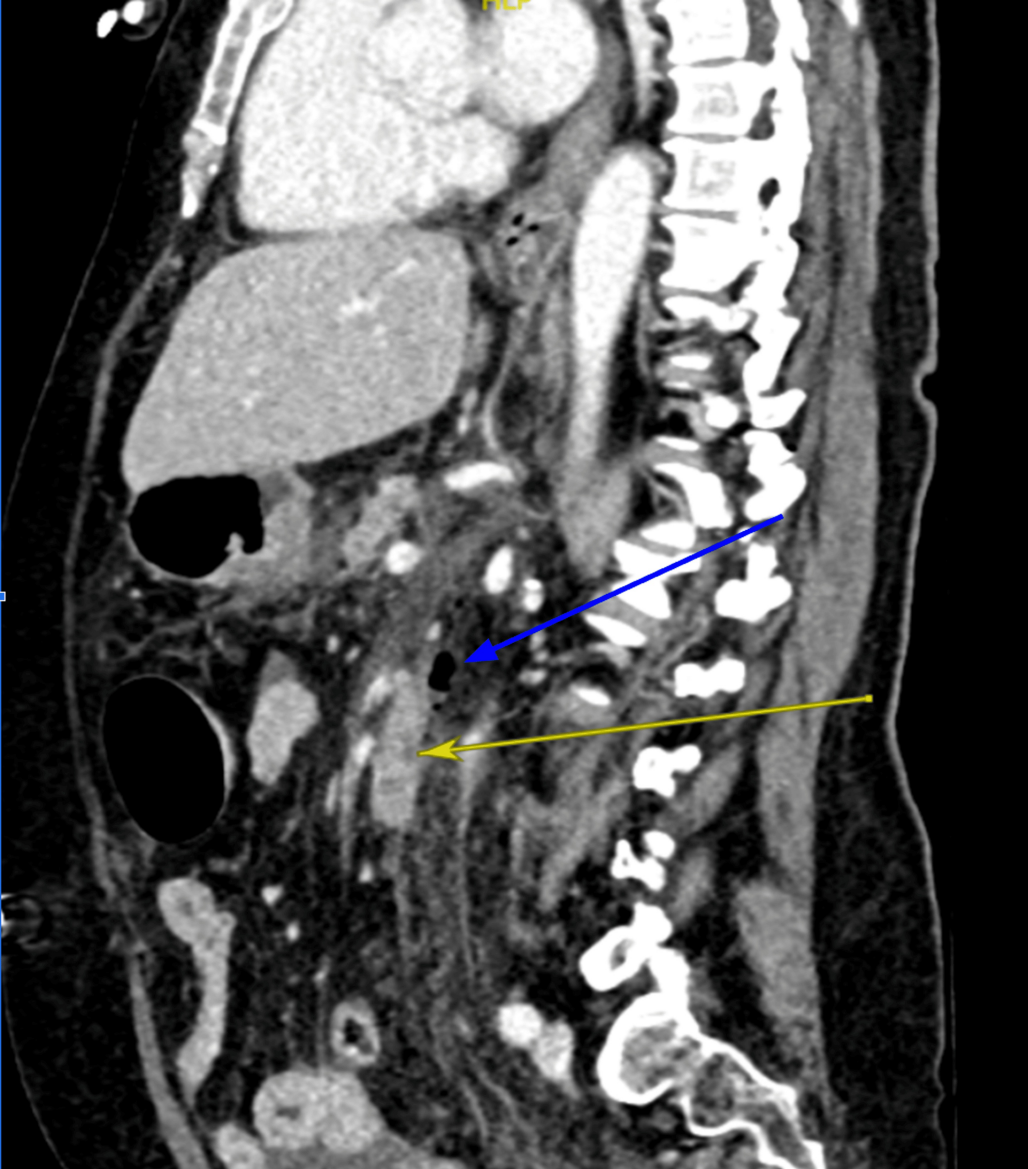
Computed tomography (CT) abdomen in sagittal view, demonstrating free intraperitoneal fluid and retroperitoneal gas adjacent to the duodenum (blue arrow) with retroperitoneal fat stranding (yellow arrow).

Initial work-up included clinical examination, showing generalised abdominal tenderness, blood investigations, and CT imaging. Blood tests (Table 1) demonstrated a markedly raised serum amylase, significantly elevated inflammatory markers, leukopenia, acute kidney injury (AKI), and coagulopathy, consistent with acute pancreatitis complicated by early shock. The diagnosis of pancreatitis was made at this stage in conjunction with biochemical evidence, particularly an elevated amylase level of 732. CT imaging demonstrated free intraperitoneal fluid and retroperitoneal gas adjacent to the second and third parts of the duodenum, raising concern for a possible duodenal perforation (Figure 2).

**Table 1:** Key laboratory parameters during the course of post-endoscopic retrograde cholangiopancreatography (ERCP) pancreatitis (PEP) leading to delayed caecal perforation. a) Initial presentation with raised serum amylase. b) Intensive care unit deterioration showing raised drain amylases and inflammatory markers. c) Low haemoglobin at the time of caecal perforation.

Parameter	Initial Presentation	ICU Deterioration	Caecal Perforation	Units	Reference Range
White cell count	1.6	18.7	13.1	×10⁹/L	4.0-11.0
Haemoglobin	153	116	65	g/L	115-165
Platelets	188	10	560	×10⁹/L	140-400
C-reactive protein (CRP)	272	266	218	mg/L	0-10
Amylase	732	36	42	U/L	30-118
Urea	10.6	7.8	4.7	mmol/L	2.5-7.8
Creatinine	117	116	40	µmol/L	60-120
eGFR	43	43	>90	mL/min/1.73 m²	>90
INR	1.8	-	1.1	-	0.8-1.2
ALT	42	50	36	U/L	10-49
Total bilirubin	6	9	8	µmol/L	0-21
ALP	63	150	142	U/L	30-130
Drain 1 amylase	-	2566	-	U/L	30-118
Drain 2 amylase	-	1582	-	U/L	30-118

Given the imaging findings and clinical deterioration, the patient underwent emergency exploratory laparotomy. Intraoperatively, a large volume of turbid fluid was found throughout the abdominal cavity with marked inflammatory changes characterised by fibrin and oedema around the duodenum. Following careful inspection, no perforation in the stomach or small or large bowel was identified. The caecum and ascending colon were dilated, but viable with no evidence of perforation at that time. Surgical drains were placed, and the patient required postoperative intensive care unit (ICU) support, including inotropic therapy and cardiorespiratory support.

Following the initial laparotomy, repeat imaging demonstrated resolution of the previously noted retroperitoneal gas, and no hollow viscus perforation was identified. In the following days, the patient developed a severe systemic inflammatory response with multiorgan failure requiring prolonged ICU management, receiving ongoing management with intravenous fluids, antibiotics, nutritional support, including sustained low-efficiency dialysis (SLED) due to anuria and ongoing AKI. During this period, analysis of surgical drain fluid on post-operative day 3 demonstrated markedly elevated amylase levels, suggestive of severe acute pancreatitis as the primary underlying pathology (Table 1).

**Figure 3:**
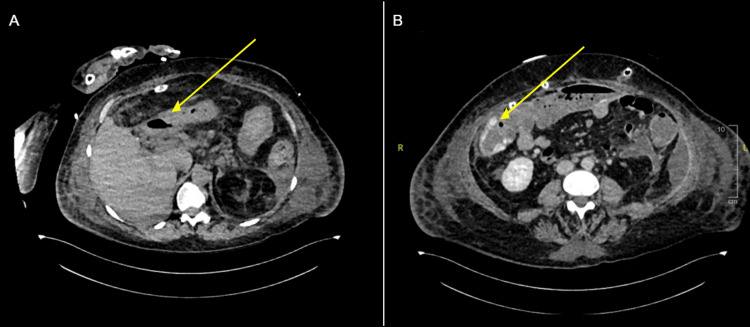
Computed tomography (CT) angiogram abdomen in axial view, demonstrating pneumoperitoneum arising from the caecum (A, yellow arrow), and active brush contract (B, yellow arrow).

Fifteen days after the initial diagnosis of PEP, during her prolonged ICU stay, the patient developed sudden episodes of melena accompanied by a drop in haemoglobin, hypotension (75/50 mmHg), and tachycardia (116 beats per minute), prompting an urgent CT angiogram to identify the potential source of gastrointestinal bleeding (Table 1). CT angiogram of the abdomen demonstrated pneumoperitoneum arising from the caecum, consistent with caecal perforation and active brush contrast indicative of active colonic bleeding (Figure 3). The patient was taken emergently to the operating room for explorative laparotomy, where a perforated caecum was identified and managed with an extended right hemicolectomy and formation of an end ileostomy.

Histopathological examination of the resected specimen obtained during the exploratory laparotomy revealed two key findings. First, there was acute transmural ischaemic necrosis of the caecum with perforation, corresponding to the site of the stoma. Second, there was saponification of the omental fat, which was in keeping with ischaemic fat necrosis typically seen in severe pancreatitis. No alternative mechanical, obstructive, or vascular cause for the caecal perforation was identified. Given the absence of another explanation, the caecal perforation was attributed to a delayed and rare complication of severe acute pancreatitis, likely mediated by inflammatory and ischaemic mechanisms.

After surgery, the patient required inotropic and ventilatory support in the ICU, was kept nil by mouth, and fed via total parenteral nutrition (TPN). She received intravenous fluids, antibiotics (piperacillin-tazobactam, vancomycin), fluconazole, and one unit of red blood cells. She was gradually weaned off inotropes and extubated. Serial CT imaging demonstrated improved intra-abdominal inflammation and resolution of collections. Later imaging demonstrated an atrophic pancreas, consistent with previous severe pancreatitis. The patient was eventually able to resume oral intake and continued rehabilitation, supported by the physiotherapy and dietetics teams, with no further evidence of intra-abdominal sepsis or biliary pathology.

This case demonstrates a rare and unexpected delayed complication of PEP, highlighting that caecal perforation can occur in the absence of other identifiable causes and should be considered in patients with unexplained deterioration during the course of severe pancreatitis.

## Discussion

Acute pancreatitis is a recognised complication of ERCP and represents the most common adverse event associated with the procedure. PEP occurs in up to 10% of cases in the general population, while gastrointestinal perforation remains rare, with an incidence of approximately 0.6% [1,2,5,10]. Importantly, when perforation does occur in this setting, it is typically related to mechanical factors, particularly stent migration, rather than pancreatitis itself causing colonic perforation. Colonic perforation due to pancreatitis is exceedingly uncommon and has been reported only rarely since its first description in 1974 [11]. Perforation arising as a secondary complication of severe acute pancreatitis reflects a distinct pathological process, driven by inflammatory and ischaemic mechanisms.

Delayed perforation of a remote colonic segment following ERCP is exceptionally uncommon. The majority of reported colonic perforations are secondary to mechanical causes, most notably biliary stent migration, direct endoscopic trauma, or pre-existing colonic pathology [7,12,13]. In the present case, no evidence of stent migration, luminal obstruction, inflammatory bowel disease, or malignancy was identified. Instead, histopathological examination demonstrated transmural ischaemic necrosis with omental fat saponification, findings that are consistent with pancreatic enzyme-mediated injury and support a secondary mechanism related to severe acute pancreatitis.

Severe acute pancreatitis is increasingly recognised as a systemic disease with significant extra-pancreatic manifestations. The inflammatory cascade triggered by pancreatic injury results in widespread endothelial dysfunction, microvascular thrombosis, and regional hypoperfusion, while concomitant retroperitoneal third spacing of activated pancreatic enzymes and inflammatory cytokines tracking along the right paracolic gutter leads to localized chemical serositis, ischaemia, and ultimately transmural necrosis and perforation of distant gastrointestinal segments [9,14,15]. Colonic involvement is a recognised but uncommon complication of severe or necrotising pancreatitis, reported in up to 15% of cases overall, encompassing a spectrum of manifestations including inflammation, stricturing, fistulation, ischaemia, necrosis, and, far less commonly, perforation [16-18]. 

Few reports have described delayed colonic necrosis and perforation developing during the second or third week of severe pancreatitis, often following an initial period of clinical stabilisation [15-19]. This temporal pattern closely mirrors the course observed in the present case, in which caecal perforation occurred approximately 15 days after the onset of pancreatitis. The delayed presentation supports the concept of progressive microvascular injury and inflammatory extension rather than an acute mechanical insult. Furthermore, the caecum may be particularly vulnerable to ischaemic injury in the setting of critical illness. Its relatively tenuous blood supply, combined with susceptibility to non-occlusive mesenteric ischaemia during periods of hypotension, vasopressor use, and multiorgan failure, has been described as a predisposing factor for caecal necrosis and perforation [19,20]. 

Diagnosis of pancreatitis-associated colonic perforation remains challenging. Clinical deterioration in severe pancreatitis is frequently multifactorial, and signs of bowel perforation may be masked by ongoing systemic inflammatory response and organ dysfunction. New haemodynamic instability, gastrointestinal bleeding, or unexplained metabolic derangement should prompt urgent reassessment and repeat cross-sectional imaging, as early recognition is critical. Management requires prompt surgical intervention for source control, alongside aggressive supportive care. Outcomes remain poor, with reported mortality rates significantly higher than those observed in uncomplicated pancreatitis, underscoring the severity of this complication.

## Conclusions

This case describes an unusual and clinically important delayed complication of PEP, with caecal perforation occurring in the absence of any identifiable mechanical cause or underlying colonic disease. The findings support an inflammatory and ischaemic process related to severe pancreatitis, rather than direct procedural injury. Notably, the perforation developed after an initial phase of apparent clinical stabilisation, highlighting that serious extra-pancreatic complications may emerge later in the disease course. In patients with severe pancreatitis who deteriorate unexpectedly, particularly with haemodynamic instability or gastrointestinal bleeding, secondary colonic pathology should be actively considered. Early reassessment with repeat imaging and timely surgical involvement are crucial, as delayed recognition may result in significant morbidity and the need for extensive operative intervention.
